# RETRACTION: Effect of Clindamycin Compared with Ampicillin‐Sulbactam as Prophylactic Antibiotics for Wound Infections Following Major Surgery for Head and Neck Cancer: A Meta‐Analysis

**DOI:** 10.1111/iwj.70191

**Published:** 2025-01-12

## Abstract

**RETRACTION**: HuY. , YanA. , and JiangF. , “Effect of Clindamycin Compared with Ampicillin‐Sulbactam as Prophylactic Antibiotics for Wound Infections Following Major Surgery for Head and Neck Cancer: A Meta‐Analysis,” International Wound Journal 20, no. 10 (2023): 4151–4158, 10.1111/iwj.14312.37483017 PMC10681415

The above article, published online on 22 July 2023, in Wiley Online Library (http://onlinelibrary.wiley.com/), has been retracted by agreement between the journal Editor in Chief, Professor Keith Harding; and John Wiley & Sons Ltd. Following an investigation by the publisher, all parties have concluded that this article was accepted solely on the basis of a compromised peer review process. The editors have therefore decided to retract the article. The authors disagree with the retraction.

**RETRACTION**: Y. Hu , A. Yan , and F. Jiang , “Effect of Clindamycin Compared with Ampicillin‐Sulbactam as Prophylactic Antibiotics for Wound Infections Following Major Surgery for Head and Neck Cancer: A Meta‐Analysis,” International Wound Journal 20, no. 10 (2023): 4151–4158, 10.1111/iwj.14312.37483017 PMC10681415

The above article, published online on 22 July 2023, in Wiley Online Library (http://onlinelibrary.wiley.com/), has been retracted by agreement between the journal Editor in Chief, Professor Keith Harding; and John Wiley & Sons Ltd. Following an investigation by the publisher, all parties have concluded that this article was accepted solely on the basis of a compromised peer review process. The editors have therefore decided to retract the article. The authors disagree with the retraction.

